# Progress in the materials science of silicene

**DOI:** 10.1088/1468-6996/15/6/064404

**Published:** 2014-12-11

**Authors:** Yukiko Yamada-Takamura, Rainer Friedlein

**Affiliations:** School of Materials Science, Japan Advanced Institute of Science and Technology (JAIST), 1-1, Asahidai, Nomi, Ishikawa 923-1292, Japan

**Keywords:** silicene, two-dimensional materials, nanoelectronics, silicon

## Abstract

In its freestanding, yet hypothetical form, the Si counterpart of graphene called silicene is predicted to possess massless Dirac fermions and to exhibit an experimentally accessible quantum spin Hall effect. Such interesting electronic properties are not realized in two-dimensional (2D) Si honeycomb lattices prepared recently on metallic substrates where the crystal and hybrid electronic structures of these ‘epitaxial silicene’ phases are strongly influenced by the substrate, and thus different from those predicted for isolated 2D structures. While the realization of such low-dimensional Si *π* materials has hardly been imagined previously, it is evident that the materials science behind silicene remains challenging. In this contribution, we will review our recent results that lead to an enhanced understanding of epitaxial silicene formed on diboride thin films, and discuss the remaining challenges that must be addressed in order to turn Si 2D nanostructures into technologically interesting nanoelectronic materials.

## 1. Introduction

**Figure 1.**
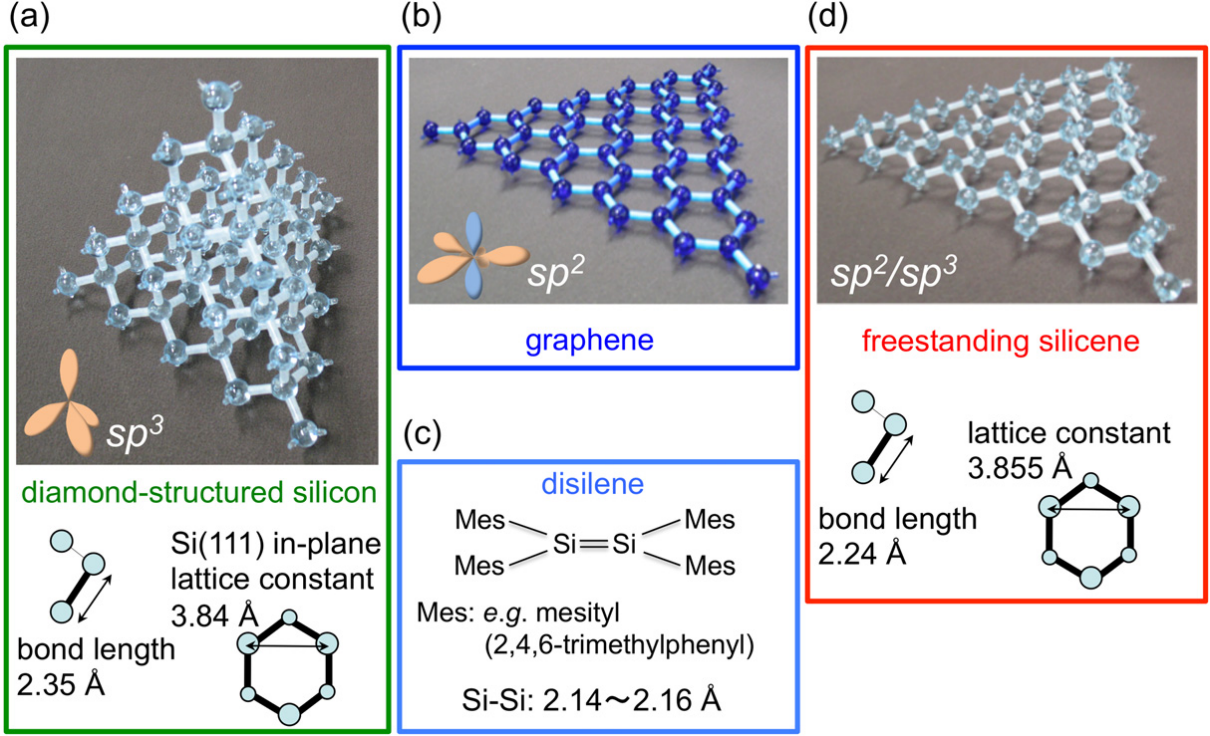
Structures and structural parameters of (a) diamond-structured silicon, (b) graphene, (c) disilene [2], and (d) hypothetical, freestanding silicene [6]. In the insets, the *sp*^2^ and *sp*^3^ types of hybridization are sketched.

Silicon is the most commonly used material in the semiconductor industry and is made of *sp*^3^-hybridized silicon (Si) atoms adopting the three-dimensional diamond structure (figure 1(a)). As a consequence, valence electrons localized in *σ* bonds are less mobile than those in graphite in which the *sp*^2^ hybridization of carbon (C) atoms (figure 1(b)) leads to the formation of extended *π* electronic states at low binding energies. Although the element Si is right below C in the periodic table, the *sp*^2^ hybridization is energetically unfavorable [1] but occurs for instance in disilene molecules [2] (figure 1(c)) and at reconstructed Si(111) surface [3]. If this type of bonding were to be realized in two-dimensional (2D) crystals made of Si atoms, the resulting material could be as exciting as the ultimately thin form of graphite: graphene. In analogy to this exciting material, an atom-thick, 2D honeycomb layer made of Si atoms possessing *π* electronic states [4] has been coined ‘silicene’ [5]. However, while the first report on a theoretical study of silicene has been published 20 years ago [6], it was only after the enormous success of graphene [7, 8] that the study of silicene-related materials has received an explosion of interest.

Already in the early study carried out by Takeda and Shiraishi [6], the crystal and electronic structures of a single, isolated or ‘freestanding’ silicene sheet (figure 1(d)) had clearly been pointed out. While silicene is the bigger cousin of graphene, there are notable and important differences and similarities between the two materials. The crystal structure of the yet hypothetical, freestanding silicene is similar to graphene: both are formed by honeycomb networks that differ, however, in the in-plane lattice constants and in the degree of flatness. In particular, while graphene is perfectly planar, silicene is predicted to be more stable when ‘corrugated’ [6], or in another word ‘buckled’ [9], in a way in which the two sublattices are displaced from each other in the out-of-plane direction. Note that originally, only the perfectly planar graphene analog has been called ‘silicene’ while the buckled form has been called a ‘Si(111) sheet’ [5].

Nowadays, the term ‘silicene’ is used to describe any 2D honeycomb structure made of Si atoms, even those in which the buckling could exhibit larger-scale periodicities [10]. Note that in its hypothetical, freestanding form, an infinite honeycomb Si sheet prefers to be buckled with two sublattices displaced from each other in the out-of-plane direction thus indeed resembling the Si(111) bilayer. While the nomination ‘Si(111) sheet’ [5] might structurally be appropriate, it does not suggest the hybridization to be different from diamond-structured silicon thus capturing the essential differences in electronic properties that make silicene special [4]. Bond lengths are indeed predicted to be shorter (2.24 Å) [6] than single bonds in the bulk of diamond-structured silicon (2.35 Å) and longer than typical but rarely occurring double bonds between Si atoms in disilenes (2.14–2.16 Å) [2]. This is indicative of a mixture of the *sp*^2^ and *sp*^3^ types of hybridizations [6]. As a result, freestanding silicene is predicted to be flatter while having a lattice constant of 3.855 Å similar to that of the Si(111) bilayer (3.84 Å) [6]. In spite of this mixture, freestanding silicene is predicted to possess *π* bands with Dirac cones at the K points [6]. The overall *π* band width is reduced as compared to graphene largely because of the smaller *π*–*π* overlap integrals between nearest neighbors which itself is related to the larger atomic radius of Si atoms [6].

More recently, as a hypothetical concept, silicene has been a productive playground with exotic electronic effects. In particular, it started to attract interest as a 2D topological insulator [11–13] and since it exhibits an experimentally observable quantum spin Hall effect (QSHE) that derives from a large effective spin–orbit coupling (SOC). The large SOC originates from the buckled nature that relates to the *sp*^2^/*sp*^3^ mixed hybridization [11]. In addition, it is large as compared to graphene, which exhibits a QSHE only at extremely low temperature [14], since the SOC scales with the fourth power of the atomic number. The SOC-induced transition to the quantum spin Hall insulator state should therefore occur at a much higher temperature as compared to graphene [11, 13]. While planar silicene is predicted to have only a vanishing gap (0.07 meV) opened by the SOC at the Dirac points, the gap opens to 1.55 meV in buckled silicene which can be further increased to 2.90 meV by applying in-plane stress [11]. Additionally, under an external, out-of-plane electric field, the two sublattices are no longer equivalent, such that the size of the band gap might be tuned [12, 13]. Eventually, by increasing the strength of the electric field, a transition from the topological insulator state to a band insulator is predicted to occur [12, 13].

Due to the predictions of graphene-like properties and those of a topological insulator emerging in a single material, the experimental demonstration of the existence of silicene has been highly anticipated. The report of the successful experimental realization of ‘epitaxial’, or lattice matching, 2D silicene sheets on metallic substrates by several groups beginning in 2012 therefore caused a stir [4, 15–18] but provoked a heated discussion on the experimental evidence related to the characteristic properties of silicene [19–22]. To sum up the present state of characterization of these epitaxial silicene phases, silicene with Dirac fermions or topological insulator-like properties has not been experimentally demonstrated with conviction. This is not surprising since quite obviously, for such epitaxial sheets on metal surfaces, it is the electronic coupling to the substrates that determines both the crystal and electronic structures of the hybrid systems. In order to reveal differences and similarities to the yet hypothetical freestanding silicene, a comprehensive characterization by a number of experimental techniques is essential and must be performed *in situ*.

Beside the basic surface science characterization, in order to explore the electronic transport properties of silicene and its potential applications in electronic or spintronic devices, the growth on insulating or semiconducting substrates, instead of the metallic substrates reported so far, is highly desired. In addition, as silicene is not chemically inert [23, 24], a capping layer is needed for *ex situ* characterizations, i.e., outside ultra-high vacuum (UHV) environments.

In this review, we summarize our recent experimental and theoretical efforts to synthesize, characterize, understand, and engineer the crystal and electronic structures of epitaxial silicene on zirconium diboride (ZrB_2_) thin films in relation to those of hypothetical, freestanding silicene, and discuss challenges in the materials science on the way towards a future silicene nanoelectronics.

## 2. The theoretical concept and the experimental realization of epitaxial silicene

### 2.1. Theoretical predictions for freestanding silicene

**Figure 2.**
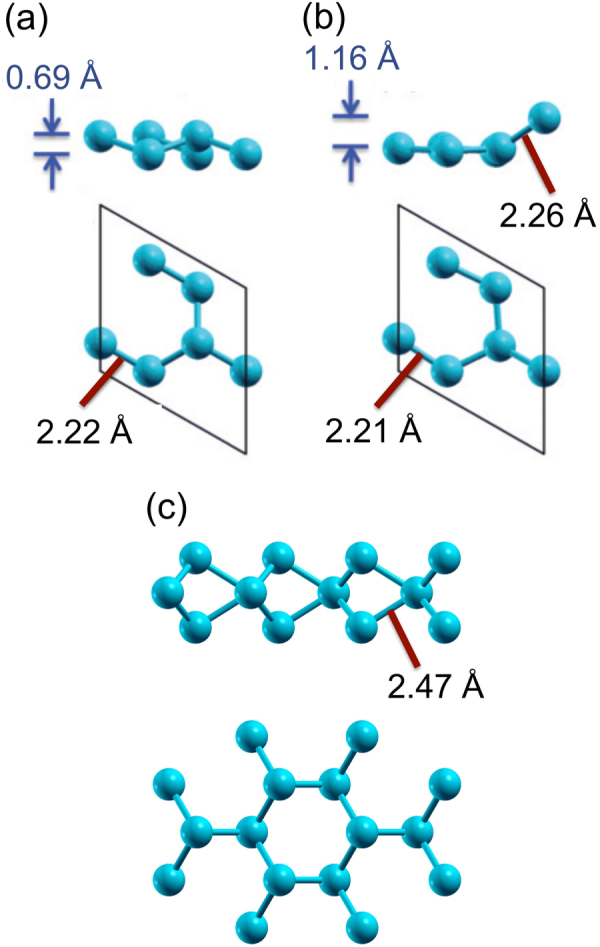
Structural configurations of freestanding, 2D Si nanostructures in side and top views: (a) regularly buckled and (b) (√3 × √3)-reconstructed, planar-like silicene phases. The bond lengths and the degree of buckling of the two phases are indicated for the calculated lattice constant of 6.35 Å that corresponds to that of the (2 × 2) unit cell of ZrB_2_(0001) surface. (c) MoS_2_-type single layer of Si atoms. Panels (a) and (b) are reproduced from C-C Lee *et al* 2013 *Phys. Rev.* B **88** 165404. Panel (c) is reproduced from F Gimbert *et al* 2014 *Phys. Rev.* B **90** 165423. Both articles published under a Creative Commons Attribution 3.0 Unported (CC BY 3.0) License.

**Figure 3.**
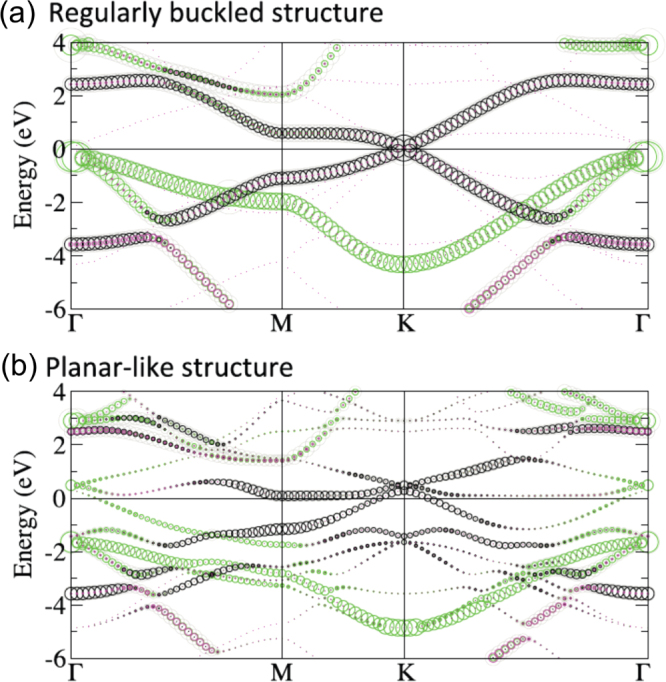
The electronic band structures of the freestanding (a) regularly buckled and (b) planar-like phases as unfolded from the (√3 × √3) unit cell (using the calculated in-plane lattice constants of the (2 × 2) unit cell of ZrB_2_(0001) surface of 6.35 Å) into the first Brillouin zone of (1 × 1) silicene. The *s* and *p*_*z*_ characters of bands are colored in magenta and black, respectively, and that of *p*_*x*_ and *p*_*y*_ orbitals in green. Adapted and reproduced from C-C Lee *et al* 2013 *Phys. Rev.* B **88** 165404. Article published under a Creative Commons Attribution 3.0 Unported (CC BY 3.0) License.

While at a first glance, the crystal structure of silicene is similar to graphene, it is not as simple as it seems. In the case of graphene, the honeycomb lattice is planar and the lattice constant remains hardly modified. On the other hand, over a large range of in-plane lattice constants, freestanding silicene is predicted to be stable in the so-called ‘low-buckled’ or ‘regularly buckled’ structure [9, 10], shown in figure 2(a), in which two sublattices of the bipartite lattice are at different heights [6, 9]. Even if regularly buckled, the atomic orbital mixing is close to that of the *sp*^2^ hybridization such that freestanding silicene is expected to display electronic properties similar to those of graphene [6, 9, 10]. As it can be recognized in the band structure shown in figure 3(a), in particular, most prominent is the absence of the opening of a recognizable gap in *π* bands at the Dirac point located at K_Si_(1 × 1). This indicates that the buckling is not associated with a gain in band energy [10] and the stability of the buckled silicene over the planar one may instead relate to an instability in the phonon part that involves the lattice repulsive potential and the response of electrons to the lattice vibration [6, 9, 10]. Of course, due to the smaller size of interatomic overlap integrals, the overall *π* band width is reduced by a factor of about 3 [5, 6, 9, 10].

Under out-of-equilibrium conditions, the degree of buckling of the honeycomb structure of freestanding silicene does vary widely as a function of the in-plane lattice constants [9, 10]. Additionally, the buckling could vary locally on the atomic length scale. This can be described as a reconstruction of the ‘original’ honeycomb, or (1 × 1) lattice. In particular, for a certain range of lattice constants, a (√3 × √3)-reconstructed phase, called the ‘planar-like’ phase [10], turns out to be quite stable. Its crystal and band structures (unfolded [25] into the (1 × 1) unit cell) are shown in figures 2(b) and 3(b), respectively.

Note that the importance of this phase lies in its relevance for epitaxial silicene: while for freestanding layers, the planar-like phase is less stable than the regularly buckled form of silicene [10], in its form with stripes [26], it becomes the ground state on the ZrB_2_ surface [10, 27]. Note that it has also been calculated to form on the Ag(111) surface [28].

In this planar-like phase, all but one of the Si atoms per hexagon reside in a single plane. In this way, it is able to sustain a longer in-plane bond length as compared to the planar phase such that the bond length becomes closer to that of the regularly buckled phase [10]. Due to the (√3 × √3) reconstruction, the symmetry is broken which causes back-folding of electronic states into the reduced Brillouin zone and the lifting of the degeneracy of bands. As can be recognized in figure 3(b), some resemblance to the band structure of regularly buckled silicene can be found. For instance, cone-like band dispersions are still noticeable even under the (√3 × √3) reconstruction, which, however, leads to the opening of a small gap and an up-shift of the corresponding features [10]. This can be visualized at the K_Si_(1 × 1) point via unfolding, as shown in figure 3(b).

Previously, the search for the lowest-energy structures of freestanding 2D Si allotropes has been focused on honeycomb lattices that relate to either regularly-buckled, unreconstructed [5, 6, 9, 10] or (√3 × √3)-reconstructed [10] silicene phases. However, very recently, it has been shown that the addition of Si adatoms to silicene results in the formation of a dumbbell structure with a lower energy per atom [29, 30]. Towards the complete coverage, the periodic dumbbells can be recognized to form the structure of a well-known single layer of MoS_2_ that possesses a lower total energy per atom than regularly buckled silicene [31]. Although hypervalent Si atoms in heteroatomic molecules have been known since the 19th century [32, 33], this came as a surprise to us since by considering the 4-fold coordination realized in the *sp*^3^ bonding of Si atoms, it is difficult to understand why bonding with 6-fold coordination could be formed by Si atoms in the MoS_2_ structure. Here, the new form of *σ* bonding expressed by three cigar-shaped orbitals coexist with an extended *π* electronic structure. The direction of these *σ* orbitals has changed from the typical in-plane direction of the orbitals in the *sp*^2^ hybridization (see figure 1(b)) to the out-of-plane direction to form cigar-shaped, so-called ‘nematic’ orbitals [31]. Quite clearly, such a previously unimagined bonding configuration and structure must be considered as a candidate for epitaxial 2D Si layers, as just predicted for the (√3 × √3) phase on Ag(111) [34].

### 2.2. The experimental realization of silicon honeycomb structures on surfaces

Honeycomb Si structures occur in disilicides [35–37], in Si sheets chemically exfoliated from calcium disilicides [38], and can even exist as a single sheet on an erbium-covered Si(111) single-crystal surface [39]. Such Si sheets in disilicides are characterized by charge transfer from the metals and a substantial amount of hybridization between the electronic states derived from the two subsystems.

More recently, Si nanoribbons with an internal honeycomb structure have been obtained by the deposition of Si atoms on the Ag(110) surface [40–44]. The epitaxial Si nanoribbons observed by scanning tunneling microscopy (STM) are uniformly 1.6 nm in width and 0.2 nm in height [40]. Density functional theory (DFT) calculation of a structure model with Si honeycombs based on STM observations resulted in an average Si–Si distance of 2.24 Å [42], a value which is close to that of freestanding silicene [6]. Along the long direction of the ribbons, dispersive states reminiscent of *π* electronic states of graphene have been observed [43]. These states are cone-like and split with a gap of 0.5 eV, centered at 0.6 eV below the Fermi level (*E*_F_) at the high symmetry point corresponding to the silicene K_Si_(1 × 1) point. In the short direction, states are localized revealing the one-dimensional (1D) character of this nanostructure [43].

The Ag(110) surface has a rectangular lattice which is ideal for the template of parallel 1D nanoribbons. On the other hand, in order to form 2D sheets with honeycomb structures, hexagonal lattices are the natural choice. Following the preparation and study of silicene nanoribbons, in 2010, the highly contested possible formation of a silicene sheet on Ag(111) surface has been reported [45]. In 2012, several groups described the formation of better-characterized Si honeycomb structures on Ag(111) [15–18] and ZrB_2_(0001) thin film [4] surfaces, of which both have hexagonal symmetry.

The deposition of Si atoms on Ag(111) single crystal surfaces under UHV conditions and in the typical substrate temperature range between 250 °C and 300 °C leads to a number of surface reconstructions depending on the amount of Si atoms and the temperature during deposition. Since independently well-calibrated Si sources are needed to precisely establish the amount of Si atoms deposited onto the surface, the interpretation of the obtained surface reconstructions differs among these reports. As such, (2√3 × 2√3) [45] and (4 × 4) [15–17] reconstructions of the Ag(111) surface, and a reconstruction corresponding to a (√3 × √3)-reconstructed silicene lattice [18] have been reported as silicene sheets on the Ag(111) surface. These reconstructions may represent various phases of epitaxial silicene or alternatively, as has been discussed, of Si networks that consist of incomplete honeycombs [18] or possibly bilayer [46] structures.

It is obvious that control of the source conditions and of the temperature is key to the formation of silicene phases by a deposition process. The advantage of this process is that different substrate materials can be easily tested. This lead to the reports on Ir(111) [47] and ZrC(111) [48] as possible substrates.

On the other hand, epitaxial silicene on ZrB_2_(0001) thin films forms by a completely different process: surface segregation at elevated temperatures. We found that on the surface of oxide-free, single-crystalline ZrB_2_ thin films grown epitaxially on Si(111) wafers, epitaxial silicene forms spontaneously [4]. The thin films of metallic ceramic zirconium diboride, with the thickness of about 15–30 nm, have been grown by UHV-chemical vapor epitaxy and exhibit the epitaxial relationship described by ZrB_2_(0001)//Si(111) and ZrB_2_[101̄0]//Si[112̄] [49].

In the case of surface segregation, the source of Si atoms is the silicon substrate of the diboride film. The process is governed by thermodynamic equilibrium conditions and thus self-terminating. Following the transfer under ambient conditions, native oxides including those containing Si atoms formed upon exposure to air are easily removed by heating to 800 °C under UHV conditions such that the diboride surface is again spontaneously and uniformly covered with epitaxial silicene. Importantly, under optimal conditions, this procedure highly reproducibly leads to samples with more than 99.5% of the surface covered with ZrB_2_(0001) terraces, and thus with single-crystalline-like silicene [49, 50]. Different from the deposition technique, surface segregation processes are materials specific and are thus not applicable to any kind of substrate. But for example, it is known that on AlN(0001) epilayers grown with Si-doping or on Si-containing substrates, Si-induced surface reconstructions are observed [51]. Here, surface-segregated Si atoms work as a surfactant [51] just like in the case of ZrB_2_ thin films grown on Si wafers. If a comprehensive characterization would be applied to this surface, one may possibly find epitaxial silicene on an insulating substrate.

### 2.3. The decisive role of a comprehensive characterization of epitaxial silicene phases

As many controversial discussions of early results have shown, evidence for epitaxial silicene should come from a combination of comprehensive experimental characterization techniques and first-principles calculations covering both structural and electronic properties. STM performed in UHV is the most frequently used characterization method for epitaxial silicene [4, 15–18, 45, 47], and in the most cases [15, 16, 18, 45, 47], combined with an electron diffraction technique, the only characterization method. But clearly, STM images alone are not enough to claim the formation of silicene since it provides limited information about the electronic structure of the surface layer.

For instance, in our original [4] and subsequent studies [27, 52–54], structural evidence for epitaxial silicene on diboride thin films grown on Si wafers has been obtained from atomic-resolution surface imaging using STM, chemical information such as on the elemental composition and the chemical environment of Si atoms from high-resolution, core-level photoelectron spectra measured at a synchrotron radiation facility, and band structure-related information from the combination of angle-resolved photoelectron (ARPES) spectra and DFT calculations. Complementary and additional structural information might be obtained for instance by reflection high-energy electron diffraction (RHEED) [49], low-energy electron diffraction (LEED) [52] and from the analysis of incident electron beam energy dependent intensities (*I*–*V*) of LEED spots [55].

Why so many characterization techniques are needed is because (i) there is no single, reliable identification method for epitaxial silicene, and (ii) epitaxial silicene phases are always in a kind of hybrid state with the surface of the respective substrates and thus not well-defined in terms of isolated, freestanding or ‘ideal’ silicene. Since epitaxial silicene is not stable under ambient conditions, the characterization must be performed *in situ* inside the UHV set-up accompanying the preparation of silicene. In order to then obtain the required variety of meaningful data, the samples should reproducibly be synthesized in different experimental set-ups. This makes experimental research very challenging and that is why the number of groups working on the synthesis and characterization of epitaxial silicene is small compared to those who are carrying out theoretical studies.

## 3. Evidence for epitaxial silicene on zirconium diboride thin films

### 3.1. Surface reconstruction and large-scale stripe formation

**Figure 4.**
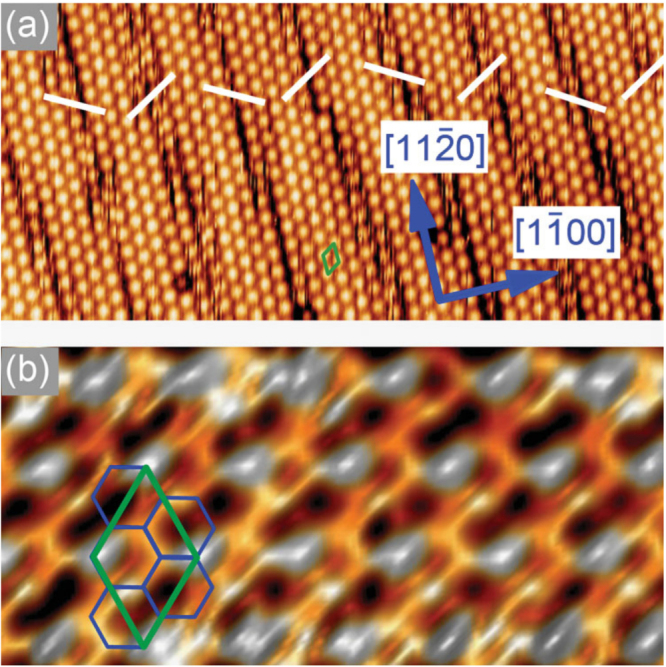
STM images of the (2 × 2)-reconstructed ZrB_2_(0001) surface with different length scales: (a) 20 nm × 9.5 nm, (b) 4.2 nm × 2 nm. The white lines emphasize the direction of offsets between successive domains. The (2 × 2) unit cell and the honeycomb mesh are emphasized by green and blue solid lines, respectively. Reproduced from A Fleurence *et al* 2012 *Phys. Rev. Lett.* **108** 245501. Article published under a Creative Commons Attribution 3.0 Unported (CC BY 3.0) License.

Figure 4(a) shows the typical large-scale STM image of epitaxial silicene observed as a (2 × 2) surface reconstruction of ZrB_2_(0001) thin films grown on Si(111) wafers [4]. For the first time, this reconstruction has been observed by us nearly 10 years ago [56], but it was not until recently and only in combination with the use of additional characterization methods and calculations that we were able to relate this surface structure to an atom-thick, buckled honeycomb structure made of Si atoms sitting on top of the diboride surface.

Under good experimental conditions, fine details can be recognized in constant-current STM images as shown in figure 4(b) [4]. The fine details are related to both the structure of the layer [4] and electronic properties [53]. It is understood that the layer corresponds to a honeycomb mesh with the lattice constant of about 3.65 Å. The layer is compressed by 5% with respect to a bulk Si(111) bilayer, and is thus similarly compressed with respect to hypothetical, freestanding silicene. It is (√3 × √3)-reconstructed such that its unit cell is adjusted to that of the ZrB_2_(0001)-(2 × 2) unit cell [4].

Apart from the surface reconstruction, stripe domains that are offset with respect to each other are recognized [4]. The repetition of the spacing between boundaries and the alternation of the direction of the offsets are signature of the spontaneous formation of stress domains as a result of large-scale interactions within a 2D layer of these adatoms.

Very recently, the origin of the large-scale stripe pattern has been suggested by first-principles calculations [26]. In short, without stripes, the (√3 × √3)-reconstructed, one-atom-thick Si layer has been found to exhibit a ‘zero-frequency’ phonon instability at the M point. In order to avoid a divergent response, the relevant phonon mode triggers the spontaneous formation of a new phase with the observed particular stripe pattern offering a way to lower both the atomic surface density and the total energy of silicene on the particular substrate.

### 3.2. The chemical states of Si atoms

**Figure 5.**
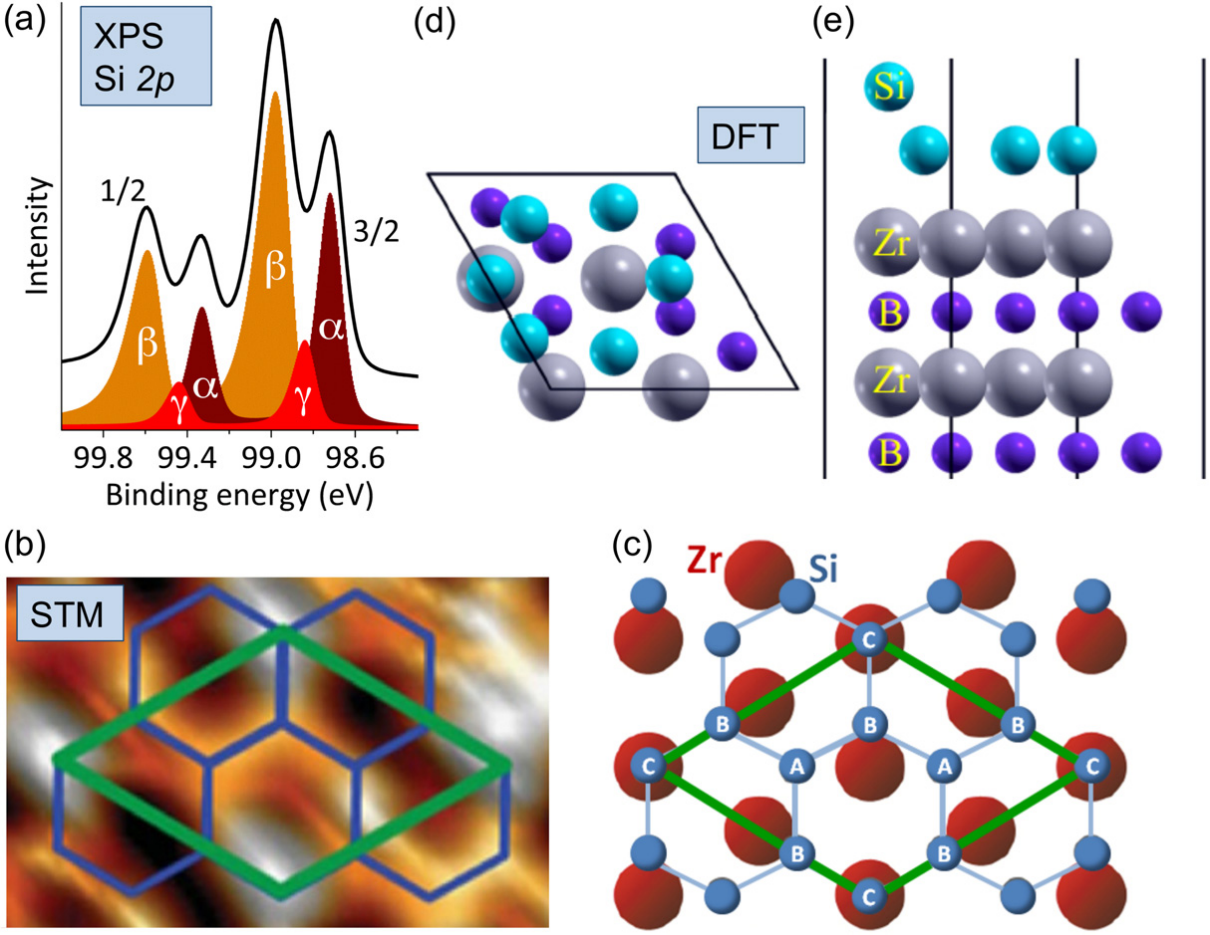
Chemical states and structural details of epitaxial silicene on ZrB_2_(0001) thin films. (a) Surface-sensitive Si 2*p* photoelectron spectrum recorded at normal emission. Chemical states identified by a peak fitting procedure are labeled *α*, *β* and *γ*. (b) STM image of the (2 × 2)-reconstructed ZrB_2_(0001) thin film surface, with the (2 × 2)-reconstructed unit cell of ZrB_2_(0001) shown in green and the model honeycomb lattice shown in blue. (c) Model of the Si honeycomb structure on the topmost Zr layer of ZrB_2_(0001). Chemically different types of Si atoms ‘A’, ‘B’ and ‘C’ are indicated. (d) and (e) Calculated structure of (√3 × √3)-reconstructed, planar-like silicene on the Zr-terminated ZrB_2_(0001) surface, as seen in the top and side views, respectively. Panels (a), (b) and (c) are adapted and reproduced from A Fleurence *et al* 2012 *Phys. Rev. Lett.* **108** 245501. Article published under a Creative Commons Attribution 3.0 Unported (CC BY 3.0) License. Panels (d) and (e) are adapted and reproduced from C-C Lee *et al* 2014 *Phys. Rev.* B **90** 075422. Copyright 2014 by the American Physical Society.

While STM images of the (2 × 2)-reconstructed ZrB_2_(0001) surface provide evidence for the presence of surface ad-atoms, proof for their elemental and chemical nature has been derived from Si 2*p* core-level photoelectron spectra obtained at various photon energies, *hν*, at beamlines 18 and 13 at the KEK-PF (photon factory) synchrotron radiation facility, located in Tsukuba, Japan. The surface-sensitive spectrum obtained with *hν* = 130 eV and in the normal emission geometry, shown in figure 5(a) shows the Si 2*p* doublet in which the spin–orbit splitting amounts to 600 ± 5 meV and in which each of the main lines consists of two peaks with binding energies that relate to distinct chemical environment of the Si atoms [4, 54].

The bulk 2*p*_3/2_ component of the clean Si(111)-(7 × 7) surface as assigned in [57, 58] appears at a binding energy of about 99.43 eV. The peaks associated with the 2*p*_3/2_ line of silicene are shifted by about 450–710 meV towards lower binding energy [4, 54].

The observed binding energy difference is reminiscent of the one observed between the C 1*s* electrons of diamond and graphene. Note that the C 1*s* binding energy of *sp*^3^-hybridized carbon atoms in the bulk of diamond (∼285.0 eV [59]) is about 0.8 eV higher than that of *sp*^2^-hybridized atoms in graphene (∼284.2 eV [60]). This suggests that the low Si 2*p* binding energy is related to a certain degree of *sp*^2^ hybridization for all of the Si atoms within the buckled honeycomb lattice of epitaxial silicene.

In initial attempts to identify components related to chemically distinct Si atoms in a spectrum related to chemical environments obtained with a lower experimental resolution, three components labeled *α*, *β* and *γ* have been identified by a peak fitting procedure and assigned to three chemical environments of the Si adatoms [4], denoted A, B and C in figure 5(c).

### 3.3. Structure model of epitaxial silicene

While in a wide range of in-plane lattice constants, freestanding silicene is predicted to be stable only with a single type of buckling, until now, experimentally found epitaxial silicene phases are all ‘reconstructed’ in terms of the (1 × 1) lattice. This means that the buckling varies locally on the atomic length scale in order to find ‘epitaxial’ conditions with the respective substrates. As such, the phases formed on ZrB_2_(0001) and Ag(111) surfaces differ from each other in lattice constants and reconstructions.

For silicene on ZrB_2_(0001), the existence of the three chemical environments *α*, *β* and *γ* as derived from the surface-sensitive Si 2*p* spectrum is consistent with an in-plane structure model that has been concluded from STM images (figures 4(b) and 5(b)) for the center of the stripe domains [4] accommodating the (√3 × √3)-reconstructed Si honeycomb lattice that matches the (2 × 2) unit cell of the ZrB_2_(0001) thin film surface. For this model (figure 5(c)), the Si atomic ratio is 2 : 3 : 1, among the A, B and C atomic sites. Two Si_A_ atoms per unit cell are sitting on hollow sites of the Zr lattice, three Si_B_ atoms are located at the intermediate position between top and bridge sites, or so called ‘near-bridge’ sites, and one Si_C_ atom is on top of a Zr atom.

Note that from our later work using spectra obtained with a higher experimental resolution, it has clearly been suggested that additional chemical states must be present that cannot be properly resolved by the fitting procedure [54]. The corresponding atoms may be located in extended boundary regions separating stripe domains. In particular, since on-top positions are the energetically least favorable ones [10], at the boundaries, Si_C_ atoms might be shifted away from on-top sites towards near-bridge sites [54].

While the in-plane structure model for the center of the stripe domains could be derived at an early stage of the study, the out-of-plane atomic positions related to the buckling have been proven difficult to determine experimentally e.g. from photoelectron diffraction effects observed as strong angle- [4] and photon-energy-dependent [54] intensity variations of the Si 2*p* core-level components.

While initially [4, 53], our interpretation related to the buckling of epitaxial silicene on the ZrB_2_(0001) thin film surface leaned towards a metastable, so-called ‘regularly-buckled-like’ phase, the controversial issue in favor of the theoretically preferred ‘planar-like’ structural modification [10], shown in figures 5(d) and (e), in the top and side views, respectively, has been only resolved in very recent work [27] by finding substantial agreement between the results of DFT calculations and ARPES data obtained in a wide energy range. That is, it turns out that the substrate–silicene hybrid electronic band structure can be the most useful fingerprint of the structural configuration of adsorbed Si layers, a fact that is due to the high sensitivity of electronic to structural properties. As our work shows, the agreement between calculations and the ARPES spectra has in part been achieved by slightly increasing the in-plane lattice constant. It has been pointed out that this has been necessary in order likely to account for a misestimate of the exchange-correlation energy in the generalized gradient approximation and to simulate the effect caused by the larger lattice constant of ZrB_2_ thin films and the expected lower surface density of the Si atoms induced to avoid epitaxial strain [27].

The results of the calculations and the agreement with the experimental ARPES data provide the strongest evidence so far for the presence of the so-called (√3 × √3)-reconstructed, ‘planar-like’ silicene phase on the ZrB_2_(0001) thin film surface.

### 3.4. The valence electronic structure

Since the electronic structure of epitaxial silicene is expected to be modified from that of the predicted one of freestanding silicene in terms of a modified crystal structure (that is by a different degree of buckling, by the reconstruction and different lattice constants) and by hybridization with the electronic states of the substrate surface, and also because bulk electronic states of substrate are measured as well, the interpretation of ARPES data is not so straightforward. First-principles calculations are necessary to understand the band dispersions; and to be able to calculate, one needs a structure model as a starting point.

**Figure 6.**
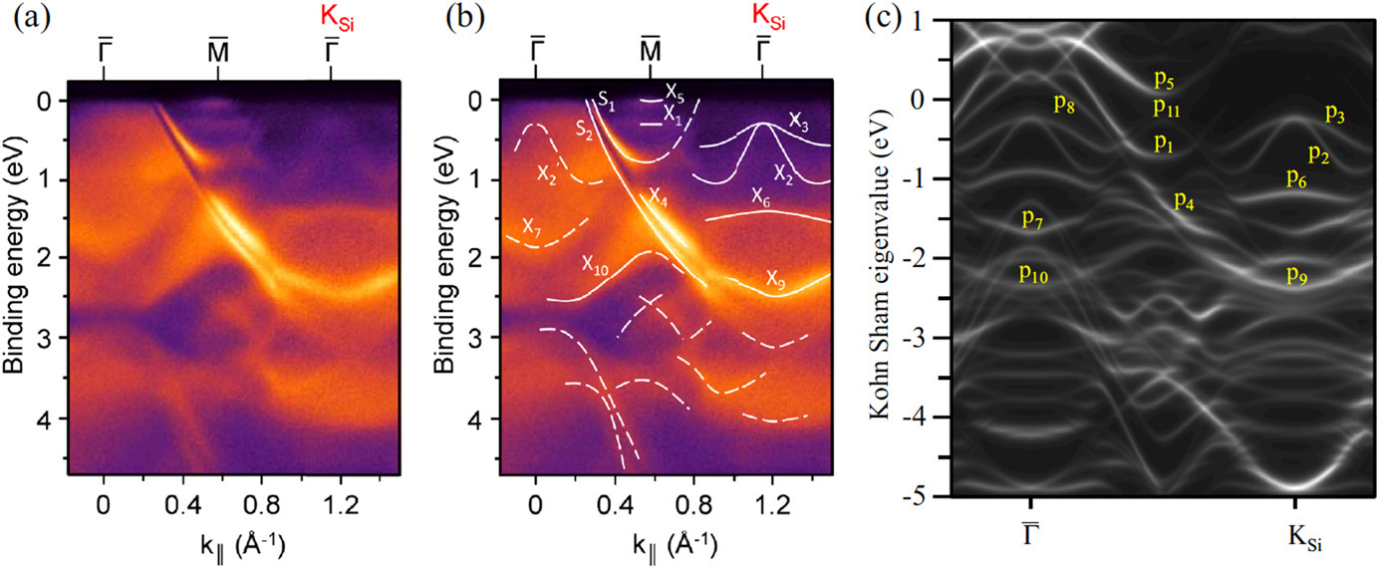
(a) ARPES spectra along the 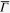
–K_Si_ direction of epitaxial silicene on the ZrB_2_(0001) surface as a function of the in-plane wave number *k*_||_. (b) ARPES spectra with guiding curves. (c) Corresponding DFT band structure of the planar-like phase. Reproduced from C-C Lee *et al* 2014 *Phys. Rev.* B **90** 075422. Copyright 2014 by the American Physical Society.

Figures 6(a) and (b) show the measured ARPES spectra along the 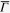
–K_Si_ direction of epitaxial silicene on ZrB_2_ thin film as a function of the in-plane wave number *k*_||_ [27]. Note that due to the (√3 × √3) reconstruction of the Si honeycomb layer, the K_Si_ and M_Si_ points of unreconstructed, hypothetical, freestanding silicene (with a unit cell containing two Si atoms) coincide with the 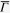 and 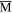 points of the repeated Brillouin zone of the reconstructed surface, respectively.

Features denoted ‘S_1_’ and ‘S_2_’ have been assigned [49] to surface states characteristic for the unreconstructed Zr-terminated ZrB_2_(0001) surface [61]. Since the Zr-derived surface states are robust, the outermost Zr layer must be considered structurally intact [4]. Other features, among those denoted ‘X_1_’, ‘X_2_’, ‘X_2_’ and ‘X_3_’, do not have a counterpart in the calculations for the unreconstructed Zr-terminated ZrB_2_(0001) surface [61] and are therefore related to the presence of silicene [4]. In particular, the intense features X_2_ and X_3_ approach *E*_F_ by up to about 250 meV at the K_Si_ point and, owing to back-folding, are mirrored as ‘X_2_’ with weak intensity in the first Brillouin zone [4, 27]. The upward curvature of X_2_ bears some resemblance to the predicted Dirac cone of *π* bands of freestanding, nonreconstructed silicene at K_Si_ [4]. The intensity changes have been discussed to be related to the sensitivity of the ARPES cross section to the selected Brillouin zone. These changes as well as the energy and dispersion of the spectral features could be represented well by DFT calculations [27].

Even if the actual stripe pattern is not accounted for, the comparison between the ARPES data and the results of the calculations shown in figure 6(c) provide a conclusive interpretation of the nature and orbital character of the states at the silicene/ZrB_2_(0001) interface [27]. It has been confirmed that all silicene-derived bands are hybridized to some extent with Zr *d* electronic states, which is consistent with non-negligible interactions at the interface. While the upward curved bands in the vicinity of *E*_F_ at K_Si_, denoted p_2_ and p_3_ (that correspond to features X_2_ and X_3_), are of partial *π* character indeed, they are actually formed by a hybridization of Si *s*, *p*_*x*_, *p*_*y*_ and *p*_*z*_ and Zr *d* orbitals. This hybridization reflects the intermediate *sp*^2^/*sp*^3^ hybridization of epitaxial silicene and its interactions with the metallic substrate [27].

Because of the presence of Zr-derived surface states and because of the hybridization of Si-derived orbitals with Zr *d* states, the surface involving silicene is metallic. However, band edges of the occupied, upward curved p_2_ and p_3_ bands, on one side, and of the downward curved band p_5_, on the other, provide a high density of states that has strong contributions within the silicene layer. Since using the ARPES technique, only the filled electronic states can be probed, the energetic separation between the band edges and thus the size of the silicene-related gap should be measured with other techniques. Scanning tunneling spectroscopy performed at temperatures as low as 5.5 K revealed a 350 meV gap, with its center shifted 60 meV below the Fermi level [53]. Although the tunneling conductance was not zero inside this gap, the observed position of band edges is consistent with the silicene-related character of the p_2_, p_3_ and p_5_ states and the close proximity of X_5_ and the Fermi level that has been observed by ARPES in doping experiments [52].

## 4. The engineering of epitaxial silicene in the light of a future silicene-based nanoelectronics

### 4.1. Doping by foreign atom adsorption

Like for graphene [62, 63], in a rigid band picture, the reaction of freestanding silicene with strong donors like alkali and alkaline earth metal atoms is expected to shift *E*_F_ with respect to the *π* bands such rendering semi-metallic silicene metallic [64, 65]. According to the predictions, potassium (K) atoms adsorb preferentially on hollow sites [64, 65]. As we have already discussed for the case of silicene on ZrB_2_(0001) thin films, the low-energy band structure of epitaxial silicene phases is different from that predicted for freestanding silicene but it is still expected that upon alkali metal adsorption, charge is donated to originally unoccupied electronic states with silicene *π* or *σ* contributions.

Recently, we have performed an experiment where small amounts of K atoms (with up to about 0.18 K atoms per Si atoms) have been deposited on epitaxial silicene formed on a ZrB_2_ thin film [52]. As the LEED pattern and even the LEED *I*–*V* have barely changed upon adsorption, it has been concluded that the type of reconstruction as well as the degree of buckling of the Si honeycomb layer remains close to that of the original one [52]. This is consistent with the prediction made for freestanding silicene in which it has been found that K adsorption does not lead to major structural changes [64].

Potassium adsorption leads to charge donation to the silicene lattice, thus n-type doping, and to the partial filling [52] of a previously almost unoccupied band, denoted ‘X_5_’, with contributions from Si *p*_*z*_ and Zr *d* orbitals [27]. Due to the charge donation, occupied states with a partial *π* character in the vicinity of the K_Si_(1 × 1) point corresponding to the unreconstructed silicene lattice shift by about 100 meV towards higher binding energy. According to a rigid band picture and n-type doping, this confirms their character as silicene-derived states.

On the other hand, while not additionally filled, a diboride surface state is affected as well which indicates an enhancement of interactions at the silicene–diboride interface upon K adsorption [52].

Since n-type doping can approximately be described by a rigid band picture, it can be expected that electron withdrawal by acceptors, e.g. iodine molecules, will lead to an upward shift of bands with (partial) *π* character.

### 4.2. Tuning of the interactions with substrates

Since epitaxial silicene phases are stabilized and since their electronic properties are determined by interactions with their respective substrates, the key for any advance towards a future silicene nanoelectronics is in the understanding of these essential interactions. So far, there are still very few cases in which epitaxial Si honeycomb lattices could be created and characterized. Most of them had been found by trial-and-error approaches or during work on unrelated topics. A more systematic approach would rely on the knowledge of the fundamental principles behind the formation mechanisms of silicene.

As already mentioned in the previous section, Si honeycomb structures have been known to exist in disilicides [35–37]. It should therefore be a matter of the strength of interactions between these honeycomb structures and the metal layers in contact if these 2D layers exhibit freestanding silicene-like properties or not. While the role of metal atoms (and in particular of *d* electronic states) for the stabilization of Si honeycomb lattices is still not fully understood, it might be expected that the change from a ‘disilicide’ picture to an ‘epitaxial silicene’ picture is gradual.

One way the interaction strength is expressed is in the hybridization between silicene and substrate electronic states. Another expression lies in differences in the thermal stability. Silicene on ZrB_2_ thin films is destabilized at temperatures above 650 °C which is observed as the ZrB_2_(0001)-(2 × 2) to (1 × 1) phase transition [66] in which fractional streaks or spots corresponding to the silicene-(√3 × √3) pattern disappear upon heating. This transition is reversible, and upon cooling, silicene ‘crystallizes’ again. A similar phase transition is observed for silicene on ZrC(111) at about 730 °C [48]. On the other hand, a silicene phase on Ag(111) observed as a Ag(111)-(2√3 × 2√3) reconstruction is known to be irreversibly destroyed upon heating beyond 330 °C [18] and also by the deposition of additional Si atoms causing a phase transition to a structure involving *sp*^3^-hybridized Si atoms and the simultaneous exposure of the Ag(111) substrate surface [67]. Obviously, interactions of silicene with the Ag(111) surface are weaker as compared to those with the outermost Zr layers of ZrB_2_(0001) or ZrC(111). This may actually be expected with regard to the tendency of zirconium forming a number of crystalline Si–Zr phases [68] while silver forms only metastable ones [69].

In the previous section, we introduced the results of K adsorption in which charge donation from K atoms to silicene has been observed. This charge donation increased the hybridization between the silicene-related and diboride-related states resulting in stronger interactions at the interface [52]. If charge donation results in stronger interaction, charge withdrawal may result in the weakening of the interactions. Further experimental efforts to engineer the interface property of epitaxial silicene by foreign atom adsorption and/or intercalation may be the key to realize silicene with properties close to those of the hypothetical, freestanding one.

### 4.3. Stability against oxidation and perspectives for capping layers

**Figure 7.**
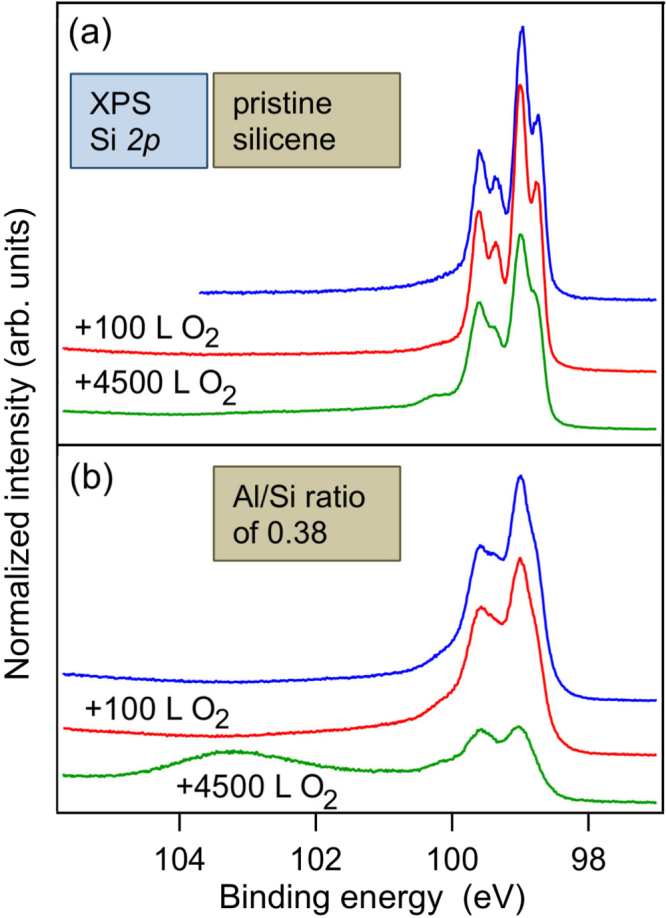
Si 2*p* spectra before and after exposure to particular O_2_ doses: 100 L and 4500 L, of (a) pristine silicene on ZrB_2_(0001) thin films and of (b) silicene covered with Al atoms at the Al/Si ratio of about 0.38. All spectra have been offset for clarity. Adapted and reproduced with permission from R Friedlein *et al* 2014 *J. Chem. Phys.* **140** 204705. Copyright 2014, AIP Publishing LLC.

Like for silicene nanoribbons [70] and silicene sheets [23], both prepared on Ag surfaces, silicene on zirconium diboride substrates resists oxidation to some extent as well. As such, while due to the presence of dangling bonds, Si(111) and Si(001) are easily oxidized, the Si 2*p* spectra of silicene on ZrB_2_(0001) thin films are hardly affected upon exposure to 4500 Langmuir (L) of molecular oxygen (O_2_) [24]. These spectra, shown in figure 7(a), have been measured at beamline D1011 of the MAX-Lab synchrotron radiation facility located in Lund, Sweden.

Still, epitaxial silicene is immediately oxidized once removed from the UHV environment. In order to be able to handle it in air, a capping layer is a must. Such layers should ideally be insulating and non-interacting, if the silicene sheet should be employed in electronics applications. Alternatively, temporary metallic capping layer could serve as protection during the transport from one UHV system to another, as long the layer can be removed at moderate temperatures.

Recently, it has been suggested that aluminum oxide (Al_2_O_3_) could be a suitable, non-destructive encapsulation material for silicene on Ag(111) [23]. However, as our study of silicene on the ZrB_2_(0001) thin film surface covered with a submonolayer of Al atoms shows, upon exposure to 4500 L, a large fraction of the Si atoms becomes oxidized (figure 7(b)) [24]. This is attributed to dissociative chemisorption of O_2_ molecules by Al atoms at the surface, producing reactive atomic oxygen species that cause the oxidation. It is concluded that aluminum oxide overlayers prepared in this fashion on silicene on ZrB_2_(0001) thin films are not suitable for encapsulation since they do not prevent but actually enhance the degradation of silicene [24].

In one of our yet unpublished studies [71], it has been found that non-destructive encapsulation of silicene on the ZrB_2_(0001) thin films by aluminum nitride deposited at elevated temperatures using trimethylaluminum and ammonia precursors is not achieved either.

### 4.4. Integration with organic electronic devices

At present, silicon-based semiconductor and organic electronics are largely separated fields. In order to achieve an integration of the two technological areas, hybrid organic/semiconductor junctions shall be created in which interfaces must be well defined and in which each of the thin films shall be crystalline. Since Si atoms favor *sp*^3^ hybridization and the formation of the diamond crystal structure, at the related semiconductor surfaces, the presence of reactive, dangling bonds leads to chemisorption of organic molecules bound with strong covalent bonds [72]. This often prevents the formation of ordered adlayers.

In our recent, yet unpublished work, the electronic and crystal structures of thin anthracene (Ac) films grown on epitaxial silicene formed on single-crystalline ZrB_2_ thin films have been studied using RHEED and ultraviolet photoelectron spectroscopy [73]. At 140 K, Ac multilayer films grow with the orientation of the long molecular axis aligned parallel to the surface. The films consist of layers in which the herringbone-stacked molecules have alternating tilt angles. The stacking distance is close to that between *bc*-planes in the Ac bulk crystal under ambient conditions [74]. The lattices of the organic film and the substrate have a commensurate relationship indicating the presence of an epitaxial relationship. This result shows that ordered multilayer films of an oligoacene molecule can be grown epitaxially on silicene.

With a low, possibly monolayer coverage, the Ac molecules also adsorb on silicene even at room temperature. This fact indicates that the interactions between Ac molecules and silicene are stronger than those on the graphite (0001) [75, 76] surface and enhanced by the atomic-site specific charge distribution associated with the buckling of silicene.

On the other hand, the chemical properties of epitaxial silicene are also quite different from those on reactive [72] Si surfaces. This enables the formation of a sharp, well-defined interface between organic molecules and silicene without the formation of covalent bonds.

In summary, the atomic-scale buckling of silicene does not prevent the growth of highly ordered organic multilayer films provided that epitaxial conditions are met. If in this way epitaxial conditions are obtained throughout device structures, highly ordered organic films can be prepared on both ultimately thin honeycomb Si layers and on widely used silicon wafers via buffer layers. The growth of ordered organic thin films on silicene may then represent a promising bottom-up approach for the integration of silicon-based and organic electronics under highly controlled conditions at various length scales.

## 5. Perspectives

With bonding configurations and properties close to but distinctively different from those of graphene, expectations for silicene are rising. Silicene is predicted to possess extremely light charge carriers with a high mobility like those of graphene in an ultimately thin sheet made of an element that dominates the semiconductor industry. The large SOC may allow the creation of perfect spin filters essential for spintronic applications [77]. Furthermore, as discussed theoretically, owing to its large surface area, silicene could serve for hydrogen storage and as a high-capacity host of lithium in Li-ion secondary batteries [78]. Silicene layers are calculated not to suffer from irreversible structural changes as *sp*^3^-type silicon anodes do, which has so far limited the commercialization of Si-based anode materials.

Although the synthesis of freestanding silicene with its exciting predicted properties remains a major challenge, and while more than two years after its experimental verification in the epitaxial form, results are limited and many properties are not fully understood, epitaxial silicene provides opportunities for the further engineering of its properties aiming practical applications. In particular, as our initial investigations show, the structural flexibility of silicene gives rise to altered electronic properties that may be tuned by applying an external stress [4]. This can be realized, for instance, through epitaxial growth on an appropriate substrate with selected lattice parameters. The introduction of a band gap through the modification of the buckling might enable the use of silicene in electronic devices that emit light or perform logical operations, something that is difficult to achieve in graphene. The gap may possibly even be tuned by an external, out-of-plane electric field [12, 13].

Given that existing Si-based technologies currently face intrinsic limits with top-down approaches, the growth of silicene with good lattice matching on large silicon wafers via buffer layers provides a way to integrate silicene nanoscale devices on silicon platforms [4]. In this context, it is essential to find ways (i) to prepare silicene on insulating substrates and (ii) for a non-reactive encapsulation in order to be able to use it outside of UHV environment. Additionally, the use of silicene in combination with ceramic buffer layers on widely used Si wafers represents even a promising bottom-up approach for the integration of silicon-based and organic electronics under highly controlled, epitaxial conditions.

While the progress in the materials science of epitaxial silicene is rapid, it remains challenging. With the promise of exceptional properties, it is clear, however, that the drive towards exciting nanoscale physics and technological applications is unstoppable.

## References

[C1] Yin M T, Cohen M L (1984). Phys. Rev. B.

[C2] Fink M J, Michalczyk M J, Haller K J, West R, Michl J (1984). Organometallics.

[C3] Pandey K C (1981). Phys. Rev. Lett..

[C4] Fleurence A, Friedlein R, Ozaki T, Kawai H, Wang Y, Yamada-Takamura Y (2012). Phys. Rev. Lett..

[C5] Guzman-Verri G, Lew Yan Voon L C (2007). Phys. Rev. B.

[C6] Takeda K, Shiraishi K (1994). Phys. Rev. B.

[C7] Novoselov K S, Geim A K, Morosov S V, Jiang D, Zhang Y, Dubonos S V, Grigorieva I V, Firsov A A (2004). Science.

[C8] Novoselov K S, Geim A K, Morosov S V, Jiang D, Katsnelson M I, Grigorieva I V, Dubonos S V, Firsov A A (2005). Nature.

[C9] Cahangirov S, Topsakal M, Akturk E, Sahin H, Ciraci S (2009). Phys. Rev. Lett..

[C10] Lee C-C, Fleurence A, Friedlein R, Yamada-Takamura Y, Ozaki T (2013). Phys. Rev. B.

[C11] Liu C-C, Feng W, Yao Y (2011). Phys. Rev. Lett..

[C12] Drummond N D, Zólyomi V, Fal’ko V L (2012). Phys. Rev. B.

[C13] Ezawa M (2012). New J. Phys..

[C14] Kane C L, Mele E J (2005). Phys. Rev. Lett..

[C15] Lin C L, Arafune R, Kawahara K, Tsukahara N, Minamitani E, Kim Y, Takagi N, Kawai M (2012). Appl. Phys. Express.

[C16] Jamgotchian H, Colignon Y, Hamzaoui N, Ealet B, Hoarau J Y, Aufray B, Bibérian J P (2012). J. Phys.: Condens. Matter.

[C17] Vogt P, De Padova P, Quaresima C, Avila J, Frantzeskakis E, Asensio M C, Resta A, Ealet B, Le Lay G (2012). Phys. Rev. Lett..

[C18] Feng B, Ding Z, Meng S, Yao Y, He X, Cheng P, Chen L, Wu K (2012). Nano Lett..

[C19] Le Lay G, De Padova P, Resta A, Bruhn T, Vogt P (2012). J. Phys. D: Appl. Phys..

[C20] Chen L, Liu C C, Feng B, He X, Cheng P, Ding Z, Meng S, Yao Y, Wu K H (2012). Phys. Rev. Lett..

[C21] Arafune R, Lin C L, Nagao R, Kawai M, Takagi N (2012). Phys. Rev. Lett..

[C22] Lin C L, Arafune R, Kawahara K, Kanno M, Tsukahara N, Minamitani E, Kim Y, Kawai M, Takagi N (2013). Phys. Rev. Lett..

[C23] Molle A, Grazianetti C, Chiappe D, Cinquanta E, Cianci E, Tallariada G, Fanciulli M (2013). Adv. Funct. Mater..

[C24] Friedlein R, Van Bui H, Wiggers F B, Yamada-Takamura Y, Kovalgin A Y, de Jong M P (2014). J. Chem. Phys..

[C25] Lee C-C, Yamada-Takamura Y, Ozaki T (2013). J. Phys.: Condens. Matter.

[C26] Lee C-C, Fleurence A, Friedlein R, Yamada-Takamura Y, Ozaki T (2014). Phys. Rev. B.

[C27] Lee C-C, Fleurence A, Yamada-Takamura Y, Ozaki T, Friedlein R (2014). Phys. Rev. B.

[C28] Chen L, Li H, Feng B, Ding Z, Qiu J, Cheng P, Wu K, Meng S (2013). Phys. Rev. Lett..

[C29] Kaltsas D, Tsetseris L (2013). Phys. Chem. Chem. Phys..

[C30] Özçelik V O, Ciraci S (2013). J. Phys. Chem. C.

[C31] Gimbert F, Lee C-C, Friedlein R, Fleurence A, Yamada-Takamura Y, Ozaki T (2014). Phys. Rev. B.

[C32] Gay-Lussac J L, Thenard L J (1809). Mém. Phys. Chim. Soc. d’Arcueil.

[C33] Holmes R R (1996). Chem. Rev..

[C34] Cahangirov S, Özçelik V O, Xian L, Avila J, Cho S, Asensio M A, Ciraci S, Rubio A (2014). Phys. Rev. B.

[C35] Baptist R, Ferrer S, Grenet G, Poon H C (1990). Phys. Rev. Lett..

[C36] Hirano T, Fujiwara J (1991). Phys. Rev. B.

[C37] Wetzel P, Sainenoy S, Pirri C, Bolmont D, Gewinner G (1994). Phys. Rev. B.

[C38] Nakano H, Mitsuoka T, Harada M, Horibuchi K, Nozaki H, Takahashi N, Nonaka T, Seno Y, Nakamura H (2006). Angew. Chem. Int. Ed..

[C39] Wetzel P, Pirri C, Paki P, Peruchetti J C, Bolmont D, Gewinner G (1992). Solid State Commun..

[C40] Leandri C, Le Lay G, Aufray B, Girardeaux C, Avila J, Davila M E, Asensio M C, Ottaviani C, Cricenti A (2005). Surf. Sci..

[C41] De Padova P, Quaresima C, Perfetti P, Olivieri B, Leandri C, Aufray B, Vizzini S, Le Lay G (2008). Nano Lett..

[C42] Aufray B, Kara A, Vizzini S, Oughaddou H, Léandri C, Ealet B, Le Lay G (2010). Appl. Phys. Lett..

[C43] De Padova P (2010). Appl. Phys. Lett..

[C44] De Padova P, Quaresima C, Olivieri B, Perfetti P, Le Lay G (2011). Appl. Phys. Lett..

[C45] Lalmi B, Ougaddou H, Enriquez H, Kara A, Vizzini S, Ealet B, Aufray B (2010). Appl. Phys. Lett..

[C46] Arafune R, Lin C L, Kawahara K, Tsukahara N, Minamitani E, Kim Y, Takagi N, Kawai M (2013). Surf. Sci..

[C47] Meng L (2013). Nano Lett..

[C48] Aizawa T, Suehara S, Otani S (2014). J. Phys. Chem. C.

[C49] Yamada-Takamura Y, Bussolotti F, Fleurence A, Bera S, Friedlein R (2010). Appl. Phys. Lett..

[C50] Fleurence A, Hubault C, Zhang W, Yamada-Takamura Y (2013). Appl. Surf. Sci..

[C51] Lebedev V, Morales F M, Romanus H, Krischok S, Ecke G, Cimalla V, Himmerlich M, Stauden T, Cengher D, Ambacher O (2005). J. Appl. Phys..

[C52] Friedlein R, Fleurence A, Sadowski J T, Yamada-Takamura Y (2013). Appl. Phys. Lett..

[C53] Fleurence A, Yoshida Y, Lee C-C, Ozaki T, Yamada-Takamura Y, Hasegawa Y (2014). Appl. Phys. Lett..

[C54] Friedlein R (2014). J. Chem. Phys..

[C55] Kawahara K, Shirasawa T, Arafune R, Lin C-L, Takahashi T, Kawai M, Takagi N (2014). Surf. Sci..

[C56] Yamada-Takamura Y, Wang Z T, Fujikawa Y, Sakurai T, Xue Q K, Tolle J, Liu P L, Chizmeshya A V G, Kouvetakis J, Tsong I S T (2005). Phys. Rev. Lett..

[C57] Himpsel F J, Heimann P, Chiang T C, Eastman D E (1980). Phys. Rev. Lett..

[C58] Miller T, Hsieh T C, Chiang T C (1986). Phys. Rev. B.

[C59] Morar J F, Himpsel F J, Hollinger G, Jordan J L, Hughes G, McFeely F R (1986). Phys. Rev. B.

[C60] Lizzit S, Zampieri G, Petaccia L, Larciprete R, Lacovig P, Rienks E D L, Bihlayer G, Baraldi A, Hofmann P (2010). Nat. Phys..

[C61] Aizawa T, Suehara S, Hishita S, Otani S, Arai M (2006). Phys. Rev. B.

[C62] Ohta T, Bostwick A, Seyller T, Horn K, Rothenberg E (2006). Science.

[C63] Bianchi M, Rienks E D L, Lizzit S, Baraldi A, Balog R, Hornekær L, Hofmann P (2010). Phys. Rev. B.

[C64] Lin X, Ni J (2012). Phys. Rev. B.

[C65] Sahin H, Peeters F M (2013). Phys. Rev. B.

[C66] Wang Z T, Yamada-Takamura Y, Fujikawa Y, Sakurai T, Xue Q K, Tolle J, Kouvetakis J, Tsong I S T (2006). J. Appl. Phys..

[C67] Acun A, Poelsema B, Zandvliet H J W, van Gastel R (2013). Appl. Phys. Lett..

[C68] Okamoto H (1990). Bull. Alloy Phase Diagr..

[C69] Olesinski R W, Gokhale A B, Abbaschian G J (1989). Bull. Alloy Phase Diagr..

[C70] De Padova P, Quaresima C, Olivieri B, Perfetti P, Le Lay G (2011). J. Phys. D: Appl. Phys..

[C71] Van Bui H, Wiggers F B, Friedlein R, Yamada-Takamura Y, Kovalgin A Y, de Jong M (2014).

[C72] Yong K S, Zhang Y P, Yang S W, Wu P, Xu G Q (2007). J. Phys. Chem. C.

[C73] Bussolotti F, Yamada-Takamura Y, Friedlein R

[C74] Brock C P, Dunitz J P (1989). Acta Crystallogr. B.

[C75] Yamane H, Nagamatsu S, Fukagawa H, Kera S, Friedlein R, Okudaira K K, Ueno N (2005). Phys. Rev. B.

[C76] Bussolotti F, Han S W, Honda Y, Friedlein R (2009). Phys. Rev. B.

[C77] Tsai W F, Huang C Y, Chang T R, Lin H, Jeng H T, Bansil A (2012). Nat. Commun..

[C78] Tritsaris G A, Kaxiras E, Meng S, Wang E (2013). Nano Lett..

